# Impact of Sex and Velocity on Plantar Pressure Distribution during Gait: A Cross-Sectional Study Using an Instrumented Pressure-Sensitive Walkway

**DOI:** 10.3390/jfmk7040106

**Published:** 2022-11-28

**Authors:** Clara Leyh, Véronique Feipel

**Affiliations:** 1 Laboratory of Functional Anatomy (LAF), Université Libre de Bruxelles, 1070 Brussels, Belgium; 2 Laboratory of Anatomy, Biomechanics and Organogenesis (LABO), Université Libre de Bruxelles, 1070 Brussels, Belgium

**Keywords:** plantar pressure, velocity, gait, GAITRite^®^

## Abstract

In-shoe systems and pressure plates are used to assess plantar pressure during gait, but additional tools are employed to evaluate other gait parameters. The GAITRite^®^ system is a clinical gait evaluation tool. Extensive literature is available for spatiotemporal parameters, but it is scarce for relative plantar pressure data. Therefore, we investigated whether, when controlling for age, the GAITRite^®^ system is able to distinguish the effects of walking velocity on plantar pressure parameters in six plantar regions in a large sample of adults. Participants (83 women and 87 men, aged 18–85 years) walked at three self-selected velocities (slow, preferred, fast) on a 6-m long GAITRite^®^ walkway. Relative peak pressure, pressure-time integral, peak time and contact area were computed for six zones (lateral and medial heel, mid- and forefoot). The impact of age (covariate), sex, side, velocity, pressure zone and their interactions on pressure variables was evaluated. Velocity affected peak pressure, pressure-time integral, peak time and contact area (*p* < 0.001). With increasing self-selected gait velocity, medial forefoot peak pressure and pressure-time integral increased (*p* < 0.001), while heel and lateral forefoot regions displayed a nonlinear plantar pressure evolution. These results suggest lower (heel strike) or more equally distributed (push-off) loads at preferred gait velocity.

## 1. Introduction

As with spatiotemporal parameters, plantar pressures are part of a battery of parameters studied in gait analysis [1]. Complementary to the clinic, they allow a functional exploration of the foot behaviour interacting with a supporting surface (floor or shoe sole) and help in the diagnosis and the prognosis of foot deformities [2,3], walking pathologies [4,5,6] and sensory disorders of the foot [7,8,9], among others.

In-shoe systems and pressure plates have unique functions and features useful for measuring, collecting, processing, and analysing plantar pressure data [1]. However, these systems often record only plantar pressures and other systems are needed to measure spatiotemporal parameters [10,11,12].

Thanks to their interconnected pressure sensor grids, some instrumented portable and flexible walkways commonly used in the study of spatiotemporal parameters also return plantar pressure distributions, and allow the extraction of data pixel by pixel and/or from defined regions of interest. Although not as exhaustive as a three-dimensional motion analysis combined with a pressure plate and a force plate, the use of such walkways is a valuable time-saver in the clinical evaluation of patients, who may not sustain long preparation periods. In addition, these systems are generally less expensive and allow individuals to walk more naturally, without restrictions [13,14,15].

As with some spatiotemporal parameters, plantar pressures vary with individual (age, height, weight, foot types and morphologies, health, joint mobility, neuromuscular activity) and environmental factors (barefoot versus shod walking, type of walking surface, etc.) [1,16]. Among the latter, increased walking velocity and cadence result in increased peak pressures at the heel [17,18,19,20], medial toes and metatarsals [13,17,18,19,20], indicating a medial shift in pressure distribution with increased velocity. In addition, increased stride length increases heel peak pressure [21] while swing and stance times tend to be negatively correlated with forefoot peak pressure. Peak time decreases in the rear- and forefoot with increasing speed and cadence [13,19,20] and interacted with changes in all temporal parameters (step time, cycle time, stride time, swing and double support time) [13]. The impact levels differ in function in the plantar anatomical areas but plantar pressure and some spatiotemporal gait components, especially gait velocity, seem to be inter-related [22].

Pressure-sensor instrumented walkways have been used extensively to study spatio-temporal gait parameters (for instance [10,11,12]). However, only a few studies [13,14,15,23,24,25,26,27] have analysed pressure distribution during gait using such systems in reasonably large samples. For these reasons, reference databases for clinical studies have not yet been established. The purpose of this study was therefore to investigate if the plantar pressure data of different regions of interest evaluated using an instrumented walkway, commonly used in the assessment of spatiotemporal parameters, show the influence of velocity in a large sample of adults of all ages.

While controlling for age, additional attention will be paid to sex and right-left plantar pressure differences. It was hypothesized that plantar pressure distribution during walking measured using the instrumented walkway (1) would correspond to the typical patterns reported in the literature [17], and (2) would vary with walking velocity and sex.

## 2. Materials and Methods

### 2.1. Participants

A total of 170 subjects (Table 1) participated in this study. Participants were eligible if they were between 18 and 85 years old. Exclusion criteria included severe orthopaedic, oncological or neurological conditions. Balance disorders that affect gait and pregnancy were also considered as exclusion criteria. All subjects were recruited from the researchers’ local community, Faculties of Medicine and Motor Sciences and from Erasme University Hospital. This study was approved by the Ethical Review Board of Erasme University Hospital and all participants signed an informed consent form prior to entering the study.

Age, sex, height and weight were reported by all subjects. In a relaxed standing and equal weight-bearing posture, the medial longitudinal arch angle of both feet was assessed, placing the centre of a goniometer at the navicular tuberosity, and the goniometer’s arms through the centre of medial malleolus and the head of the first metatarsal. Lower-limb length was determined bilaterally using a direct clinical method using a tape measure as the distance between the tip of the anterior superior iliac spine and the tip of the medial malleolus in supine position.

### 2.2. Protocol

Plantar pressure data were collected from the 6-m long GAITRite^®^ walkway (CIR Systems, GAITRite Gold v. 3.9) at 120 Hz. Each participant performed three walking trials at three different self-selected [10,11,12,13,14,15,17,23,25,26] paces (slow, preferred and fast walking velocities) following the recommendations of Kressig and Beauchet [29]. Gait speed order was randomized by lot drawing. To avoid the effects of acceleration and deceleration, participants started and ended walking two meters from walkway edges [10,11,12,25,26,29]. Before and during testing, they could practise walking across the walkway or take a break to rest.

A trapezoid mask divided each footprint proportionally into 12 zones (6 lateral and 6 medial). For each pressure zone, four variables were computed: peak pressure (maximum pressure per zone, expressed as a percentage of the overall maximum pressure), P*t (integrated pressure over time, expressed as a percentage of the overall integrated pressure over time), peak time (first time point at which one or more sensors in a zone were at the maximum level, expressed in seconds) and area (sum of the active sensor areas within a zone; expressed in square centimetres) [13]. In this study, data of the 12 trapezoids were assembled in 6 sections defined as medial and lateral fore-, mid- and hind-foot [25,26].

Average values over three trials were computed to compare the plantar pressure parameters between sides, velocities, and pressure zones.

### 2.3. Statistical Analysis

Statistics were computed in STATISTICA (StatSoft, Inc. 2007, version 8) and the level of significance was set at *p* < 0.05. The normality of all data was explored using the Kolmogorov—Smirnov test and Q-Q scatterplots. Descriptive analyses and Mann—Whitney U Test were performed on demographic characteristics (Table 1). For selected spatiotemporal parameters, a mixed-model ANOVA evaluated differences between the velocity condition, sexes and between a velocity-sex interaction (Table 2).

A mixed-model analysis of covariance (ANCOVA) was carried out to investigate univariate sex differences (between-subject factors) in pressure parameters between left and right fore- and rear-foot at slow, preferred, and fast speeds (within-subject factors) when controlling for age (covariable). Homoscedasticity and sphericity assumptions were examined with Levene and Mauchly tests, respectively. When the sphericity assumption was not met, adjusted *p*-values were computed using the Greenhouse—Geisser correction if epsilon (ε) was lower than 0.75, or the Huynh—Feldt correction if ε was higher than 0.75 [30]. Prior to the mixed-model ANCOVA, assumptions of independency of categorical independent variable and covariable as homogeneity of regression slopes were assessed using specific ANOVAs and mixed-model ANCOVAs. Tukey HSD tests were used to explore within-subject interactions and/or between-subject interactions. Partial eta-square (part. η^2^) were computed to estimate the effect size (with part. η^2^ ≈ 0.01 indicating a small effect, part. η^2^ ≈ 0.06 a medium effect, and part. η^2^ ≈ 0.14 a large effect) [30].

Mid-foot plantar pressure parameters were analysed with nonparametric statistics, as Kolmogorov—Smirnov tests were significant. Mann—Whitney U tests investigated the sex differences, Wilcoxon tests observed differences between left and right feet or medial and lateral mid-foot, and Friedman rank tests assessed the differences between velocities, sides, and plantar pressure zones. Pairwise comparisons were computed between each variable level using Wilcoxon matched pairs test. Bonferroni corrections were used to adjust for multiple comparisons in our post-hoc analysis (P_Crit_. = 0.05/k; where k is the number of comparisons tests). To convert z-scores into effect size estimates, Rosenthal’s equation was used ($r=z/\sqrt{N}$; where *r* is the effect size estimate; *z* the test statistics z-score and *N* the size of the total observations on which z is based. *r* ≈ 0.1 indicates a small effect, *r* ≈ 0.3 indicates a medium effect and, *r* ≳ 0.5 indicates a large effect) [28].

Results are reported as mean ± standard deviation or median [interquartile range]. Raw plantar pressure data, mixed-model ANCOVA, Friedman ANCOVA and Wilcoxon matched pairs test results are provided in the Supplementary Materials (Tables S1–S3).

## 3. Results

The assumptions of mixed-model ANCOVA (homoscedasticity and linearity, independency covariable-independent factor and homogeneity of regression slopes) were met.

Participant characteristics are provided in Table 1. Except for age, significant differences were found between women and men. For BMI, the observed difference was, however, trivial (*r* = −0.16). Descriptive statistics for spatiotemporal parameters and plantar pressure variables are shown in Table 2 and Table 3, respectively. Reference tables for medial and lateral plantar pressure parameters values at slow, preferred and fast velocity are displayed in the supplementary materials (Tables S4 and S5). In comparison to men, women walked slower at a fast speed with an increased average cadence, decreased average contact time and a significantly lower step length at both preferred and fast walking speed.

### 3.1. Impact of Sex

After controlling for the effect of age, male subjects presented a significantly later peak time than women in the rear- and forefoot (0.40 ± 0.01 s and 0.38 ± 0.01 s, *p* = 0.004, part. η^2^ = 0.05, respectively). In the mid-foot, the distribution of peak time did not differ between sexes (*p* = 0.11) and, whatever the statistical analysis, no differences between men and women appeared for peak pressure, P*t and area (*p* ≥ 0.23) (Table S2).

### 3.2. Impact of Side

There were no significant differences between left and right peak pressure, P*t, peak time and area in the rear- and forefoot when controlling for age (*p* ≥ 0.11). However, right mid-foot peak pressure, P*t and area medians were significantly higher than the corresponding left mid-foot medians (2.3% [1.6–3.1] versus 1.2% [0.9–1.8], *p* ≤ 0.03, r = 0.84; 4.5% [2.8–6.2] versus 3.9% [2.7–6.0], *p* = 0.01, *r* = 0.20; and 7.7 cm^2^ [5.3–10.3] versus 7.2 cm^2^ [5.2–9.9], *p* = 0.03, *r* = 0.17, respectively). No right-left differences were noted for mid-foot peak time (*p* = 0.07).

### 3.3. Impact of Velocity

When controlling for age, rear- and forefoot peak pressure and P*t varied with velocity changes (*p* < 0.001, 0.28 ≤ part. η^2^ ≤ 0.30), area increased with increasing velocity (*p* < 0.001, part. η^2^ = 0.26) while peak time decreased from slow to fast speed (*p* < 0.001, part. η^2^ = 0.30). In the mid-foot, peak pressure, P*t, peak time and area decreased with increasing velocity (*p* < 0.001, 0.46 ≤ *r* ≤ 0.95) (Table 3).

### 3.4. Sex, Side and Velocity Interactions on Overall Plantar Pressure Data

When controlling for age, a side-velocity interaction was observed for rear- and forefoot peak pressure (*p* < 0.001, part. η^2^ = 0.08) revealing a significant increase of left peak pressure especially marked at the fast speed. In contrast, in the mid-foot and mainly at slow speed, left peak pressure was significantly lower in comparison to right peak pressure (*p* < 0.001, *r* = 0.79). This reflected also in the left-right mid-foot P*t results at slow speed (*p* = 0.004, *r* = 0.22).

Moreover, peak time was influenced by a side-velocity-sex interaction when controlling for age (*p* = 0.004, part. η^2^ = 0.04) since, at slow speed, women presented significantly delayed right rear- and forefoot peak time (.51 ± 0.02 s) in comparison to women and men left peak time (0.50 ± 0.02 s and 0.55 ± 0.02 s, respectively).

Area did not present a significant interaction effect between side and velocity and/or sex on overall plantar pressure data (*p* ≥ 0.34).

### 3.5. Pressure Zones and Interactions with Velocity

**Figure 1:**
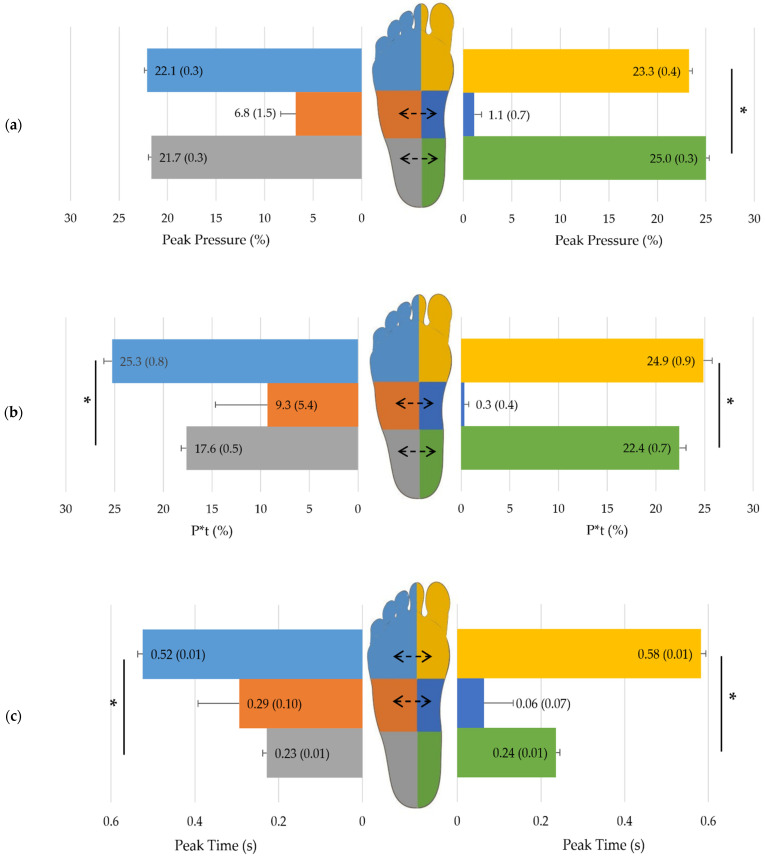

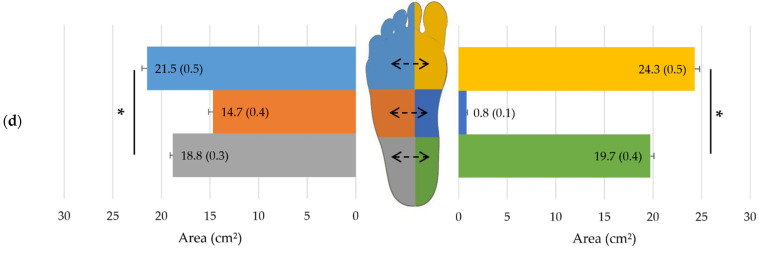
(**a**) Peak pressure, (**b**) P*t, (**c**) Peak time and (**d**) Area mean (and standard error) [computed for covariates at their means: age = 42.5 yrs] of each specific pressure zone; computed on all subjects (*n* = 170). Asterisks (*) denote statistical significances between forefoot and rear-foot plantar pressure zones in the lateral or medial part of the foot. Significant differences between lateral and medial pressure zones of fore-, mid- and rear-foot are indicated by dashed arrows in the drawn footprint (*p* < 0.05).

**Figure 2:**
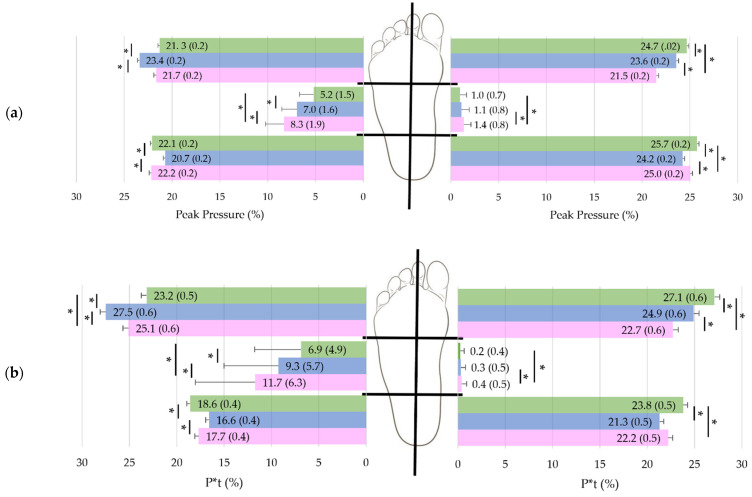

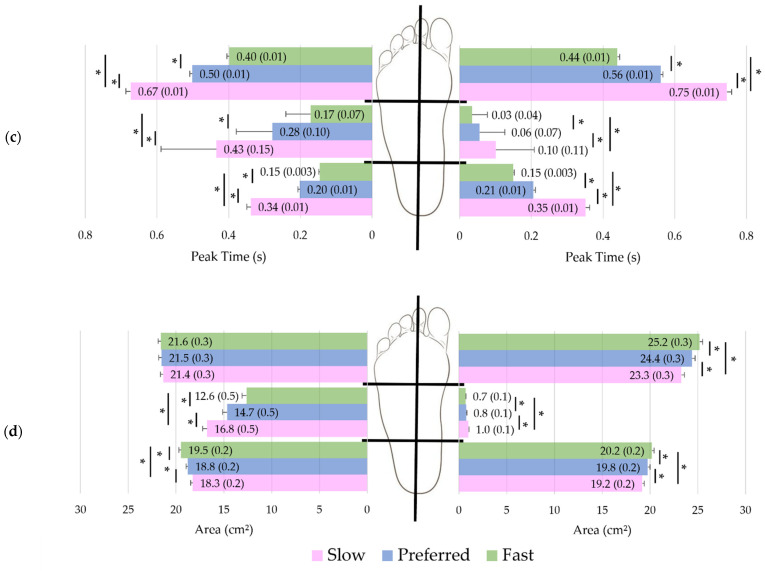
For each define pressure zone, (**a**) Peak pressure, (**b**) P*t, (**c**) Peak time and (**d**) Area mean (and standard errors) [computed for covariates at their means: age = 42.5 yrs] at each specific walking speed (from bottom to top): slow velocity results are in pink, preferred in blue, and fast in green. Results are computed on all subjects (*n* = 170). Asterisks (*) denote statistically significant differences between velocities within each plantar pressure zones (*p* < 0.05).

Peak pressure, P*t, peak time and area differed between plantar pressure zones (*p* ≤ 0.02; in the mid-foot, 0.61 ≤ *r* ≤ 0.87 and, in the rear-/forefoot regions, 0.03 ≤ part. η^2^ ≤ 0.86) and reflected (1) typical double-peak plantar pressure and contact area distribution patterns, (2) increased total pressure exposure in the forefoot, and (3) timing progression during foot rollover (Figure 1). Furthermore, velocity impacted heterogeneously these parameters within the plantar pressure regions (*p* < 0.001; in the mid-foot, 0.83 ≤ *r* ≤ 0.89 and, in the rear-/forefoot regions, 0.03 ≤ part. η^2^ ≤ 0.14) (Figure 2 and Tables S4 and S5).

Except in the mid-foot where no differences in peak pressure were noticed between preferred and fast velocities (*p* = 0.05), all other plantar pressure zones presented different peak pressure with a decrease or increase in walking velocity (*p* ≤ 0.04) (Figure 2).

With increasing velocity, medial forefoot peak pressures increased (*p* < 0.001) while lateral mid-foot peak pressure decreased (*p* < 0.001). Medial and lateral heel peak pressures were smaller at preferred walking speed compared to both slow and fast velocities (*p* ≤ 0.04) while, in the lateral forefoot, a reduced peak pressure at slow and fast velocities was observed in comparison to preferred speed peak pressure (*p* < 0.001). At the medial mid-foot, peak pressure was higher at slow compared to preferred and fast velocity (*p* < 0.001) (Figure 2).

P*t evolved with walking velocity, as did peak pressure (*p* ≤ 0.02) except that there was no difference in medial heel P*t between slow and normal walking speed (*p* = 0.09). Strongly dependent on walking speed, peak time decreased with increasing walking speed in all studied plantar pressure regions (*p* < 0.001). Finally, with increasing speed, contact area increased in the medial forefoot and heel regions (*p* ≤ 0.008) and decreased in the mid-foot zones (*p* ≤ 0.004). Lateral forefoot contact area was not influenced by walking velocity (*p* ≥ 0.99) (Figure 2).

### 3.6. Influence of Sex on Pressure Zones and Interactions

**Figure 3:**
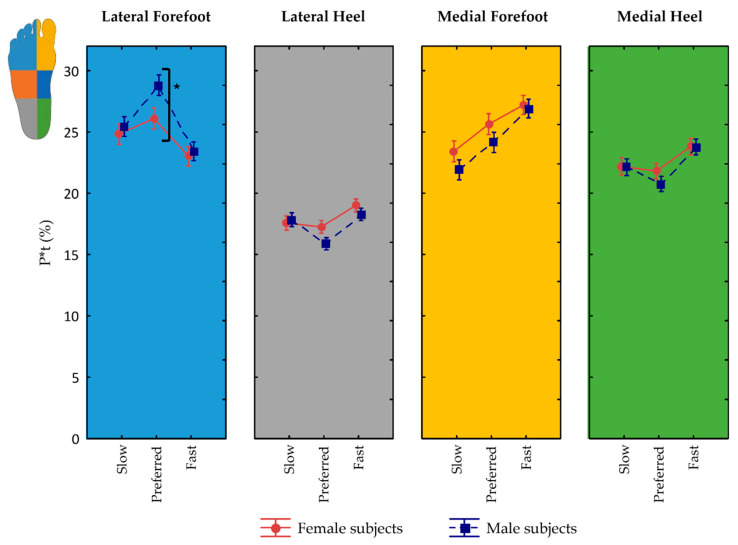
For lateral and medial fore- and rear-foot, P*t in men (*n* = 87) and women (*n* = 83). Asterisks (*) denote statistically significant differences between women and men in specific plantar pressure zones at each gait speed (*p* < 0.05).

At preferred velocity in the lateral forefoot, men presented greater P*t than women (*p* ≤ 0.04). No further differences were observed between women and men when comparing equivalent pressure zones at specific walking velocities (*p* ≥ 0.07) (Figure 3).

However, the effect of velocity on peak pressure and P*t in the different plantar pressure zones was mainly observed in male participants (*p* ≤ 0.04, part. η^2^ = 0.02) where greater variation in peak pressure and P*t with increased or decreased walking speed were present. Instead, women showed increased peak pressure and P*t in the medial forefoot only when they increased walking speed from slow to preferred (*p* ≤ 0.006). Moreover, greater peak pressure in the lateral heel (*p* = 0.04) and reduced P*t in the lateral forefoot (*p* < 0.001) were observed only at fast speed.

Furthermore, peak time was shorter in female subjects in both lateral and medial forefoot regions (*p* ≤ 0.01, part. η^2^ = 0.09).

## 4. Discussion

Whereas the GAITRite system is commonly used for the analysis of spatiotemporal gait parameters, its application in the field of plantar pressure distribution assessment during gait is scarce [13,14,23,24,25,26,27]. Previous studies did not aim at establishing a reference database which takes into account a sufficiently large sample (maximal control group of 62 subjects [13]), nor was the effect of gait speed considered (participants walked at a single preferred gait speed). The present study can thus be considered to be a solid normative reference for future clinical applications, as studies have shown alterations of pressure distribution during gait during pregnancy [25,26], and in patients with different disorders, such as diabetes [24], low-back pain [23] and autism [14]. The database presented in this work can contribute to facilitate the use of the GAITRite system to assess relative pressure distribution in the clinical context of diagnosis and follow-up.

The recorded plantar pressures measured in this study reflected the typical plantar pressure pattern and their value conformed to those described in the literature [13,14,17,23]. The pattern also varied with walking speed (slow, preferred, fast), which, along with the other reported spatiotemporal parameters, reproduced known sex differences. These parameters were slightly lower but within the normal limits described in the literature [31,32]. The slow and preferred velocities reported by Rosenbaum et al. [17] corresponded to those of our study. These authors observed that by increasing the walking speed, young subjects showed an increase in peak pressure at the heel and medial forefoot (related to hallux, 1st metatarsal and 2nd–3rd metatarsal anatomical areas). On the other hand, peak pressure in the lateral forefoot decreased with increasing velocity. Gait speed also negatively influenced the local impulse (aka P*t) in the heel where, when speed increased, the hind-foot contribution to the total loading of the foot was reduced, even if peak pressures were higher.

In our study, peak pressures by area showed a similar evolution from preferred to fast speed. At slow speed, however, lateral forefoot and heel peak pressures and P*t were close to those at fast speed, displaying a U-shaped variation with velocity. This finding differs from the evolution traditionally described in the literature [17,18,19]. However, Rosenbaum et al. [17] showed an identical P*t speed evolution pattern at the medial heel and the central forefoot but did not emphasise or explain this curvilinear evolution.

Moreover, for each foot region studied, Taylor et al. [19] did not demonstrate differences in pressure peak or P*t between slow and preferred speeds, indicating a non-linear evolution of plantar pressures within toe 2, toes 3–5, lateral mid-foot and hallux metatarsophalangeal joint with increasing velocity. However, within our sample, peak pressures and P*t were significantly different between slow and preferred velocities. This could be due to the speeds observed as slow and preferred, which were different between our studies. The slow speed in Taylor’s study [19] (1.10 m/s) was close to our preferred speed (1.25 ± 0.20 m/s). Secondly, the difference between reported slow and preferred velocities (56% in our study versus 28% in Taylor’s study [19]) may also explain the different results, as could the different masks applied to the footprint. The GAITRite system uses a mask that is less specific from an anatomical point of view.

In the medial, central and lateral forefoot (without inclusion of the toes), Segal et al. [18] established an initial increase of peak plantar pressures with increasing velocity, followed by a plateau or even a decrease at the fastest speeds (above 1.5 m/s in the central and lateral forefoot). This finding is similar to the lower lateral forefoot peak pressure found in our study at fast velocity and illustrates that, with velocity changes, quadratic models appeared to fit best peak pressure evolution in these regions. On the other hand, heel and hallux peak pressure evolved linearly with increasing speed [18]. The differences observed between studies at the heel and medial forefoot could be clarified by the measuring device (pressure insoles), the imposed gait velocities, the use of a treadmill and the applied mask [18].

Finally, similar to the relationship between walking velocity and oxygen consumption [33,34] or lower-limb kinematics variability [31,35,36], the average peak pressure and P*t data computed with the GAITRite walkway displayed a U-shaped evolution with increasing velocity in the lateral forefoot and hind-foot. An “optimal” velocity, also known as “economic” [33,34,37], could be defined as the speed at which the peak pressure and the P*t are lowest and could match the individual preferred walking speed. This velocity could reduce the loads on the heel and forefoot, respectively, during heel strike and push-off.

At fast speed, the increased heel peak pressure and P*t can reflect an increase in ground reaction forces generated during loading response phase [38,39]. The ground reaction forces are opposed to the downwards momentum induced during initial contact [39], increasing consecutively the dynamic load on the musculoskeletal system [40]. At the same time, the muscles are activated to increase the centre-of-mass vertical acceleration and anterior propulsion [41,42]. Velocity-induced changes on spatiotemporal characteristics, such as increased step and stride length, also generate increased muscle activity to allow the swing phase of gait [43]. In addition, the boat-shaped foot structure, as well as the link between the triceps sural, Achilles tendon, flexor digitorum (and hallucis) brevis and plantar fascia [44,45,46], make it possible to store the forces accumulated during the early support phase and to avoid longitudinal arch collapse [47]. This induces in particular a decrease in pressures (peak and P*t in our study) at the midfoot and forefoot [47]. With regard to the optimal walking speed, simulation modelling data showed that the elastic energy stored and returned reached a maximum of 1.2 m/s [48]. On the other hand, with increasing speed, the decrease in peak pressure and P*t observed in the lateral forefoot at fast speed may reflect a medial shift in the centre of pressures [49,50].

Since at slow speeds the ground reaction force is less than the gravitational force, additional muscular activity may be required to raise the centre of mass during the initial contact phase [37]. The potential increased muscular forces at the hind-foot could explain the increased pressure, reflecting the lower mechanical efficiency of slow speed (deviation from the natural frequency of pendulum movement [37,51] and increased variability [35]). In addition, walking at slow speed is less conducive to the storage and recovery of elastic energy in the musculotendinous complex [51], which can induce in particular a lower ankle push-off.

As this study did not include a kinematic and kinetic analysis of ground reaction forces, centre of pressure excursion and foot type or foot angle progression, these remain hypothetical explanations.

No differences between men and women were highlighted in the relative peak plantar pressures data and contact area, while a difference in body weight and foot height was present (Table 1). However, an increased peak pressure and P*t in the heel and medial forefoot of men and in the medial mid-foot of women as well as a larger contact area in men compared to women have been identified in the literature [12,52,53]. These differences are likely driven by differences in foot structure, shape and size [54], vertical centre of mass displacement, body weight [55], ligament laxity and joint stiffness [56,57] between sexes.

Our study detected men-women differences in medial and lateral forefoot peak times as well as lateral forefoot P*t at preferential speed. With an equivalent average speed between men and women, these differences could reflect a coupled disparity in contact time, step lengths [21] and cadence observed between men and women in our study (Table 2). Titianova et al. [13] demonstrated, indeed, that peak time increased with the decrease in cadence (lower in men than in women in our study) in the forefoot and that it was strongly related to the temporal gait parameters. They demonstrated further that subjects with higher body weight (such as the group of male subjects in this study) had delayed peak times. Body mass was also an important determinant of peak pressure in the lateral forefoot, as in all other regions of the foot, except the heel and hallux [22]. Finally, men had a higher P*t than women in the lateral forefoot, which may reflect an increase in average pressure and/or contact time in this region due to a longer step length [21]. Indeed, when stride length increased, Allet et al. [21] observed that P*t increased at metatarsals 1 and 5, mid-foot and under toes 3 to 5 and decreased at heel, hallux, second toe and metatarsals 3 and 4.

In the women’s samples, plantar pressures distribution seemed to be less sensitive to the effects of walking speed, although peak pressure was increased at fast speed at the lateral heel, probably under the influence of cadence. Indeed, women tend to increase their gait speed by increasing the pace [31]. Furthermore, the centre of pressure trajectory, through the stance phase of gait is more medially distributed in women than in men [58]. This may explain the possible increase in pressure and P*t in the medial forefoot (as in [53]) and the decrease in lateral forefoot peak pressure observed in women. Conversely, men seemed to distribute the load over the entire forefoot.

Finally, the important disparity between studies (protocols, measurement devices, footprint masks and units of measurements) prevent an effective comparison of the results [59] and is potentially the cause of divergent outcomes between studies, such as those concerning the impact of age [12,60,61,62,63]. For instance, midfoot peak pressure was reported to have increased [61], decreased [60] or be unchanged [62] in elderly adults. Plantar pressures changes during gait in the elderly are declared to be influenced by several factors, such as plantar callosity formation [60], loss of fat pad elasticity [61], toe deformation [62], a decrease in muscle strength [62], an increase in step width [60], centre of pressure medialisation [60], among others, indicating a less propulsive gait pattern [60,62]. These differences justify the choice of the integration of age as a covariate in this study.

These findings should, however, be considered in the light of several limitations, as the use of self-selected speeds did not allow a controlled walking speed. The latter provide advantages for normalizing individual differences, comparing subjects and eliminating the effects of gait velocity on plantar pressure distribution. However, in the perspective of clinical applications, self-selected speed protocols are part of consensual guidelines [29] and are considered valid, reliable, time and cost effective and representative of the patient’s capacities [64]. Future work should include stratification strategies to better represent age-distribution through the study sample.

Due to the high intra-subject variability observed by some authors [65,66,67], recording a large number of steps (>500 steps per individual) is recommended to represent the variability and central tendency of individual plantar pressure characteristics with a high degree of accuracy [65,66]. However, some authors claimed that only 4 to 20 steps are required to achieve acceptable levels of reliability [68,69,70]. The recording of more than 500 footprints is only reasonably feasible in samples of healthy individuals. Subjects with pathologies would indeed need to walk for more than five min (and this for each walking speed) in a laboratory environment in order to reach this number of steps. A treadmill offers this possibility more easily than a walkway or platform. Indeed, on a system such as the 6-m long GAITRite, about 60 walks should be performed in order to record up to 500 steps. In protocols where several velocities are assessed, this is not realistic with a patient. In our study, participants walked over a distance of approximatively 18 m (3 × 6 m walking distance) and between 20 and 30 steps were recorded per walking velocity, which is higher than most reports in the literature [18,67]. Finally, by allowing a continuous recording of several footprints during walking, the GAITRite system offers research opportunities to deepen the understanding of intra-individual variability observed within plantar pressures.

In addition, the pressures applied on the walkway (GAITRite or similar) are transformed into relative pressures on a scale of six switching levels over which researchers and clinicians have no control. This plantar pressure data presentation is linked to the impossibility of calibrating the number of sensors included in the active surface of the walkway. As a consequence, pressure variables are not presented as absolute values. Although this approach can be criticised and makes comparisons with the literature difficult [59], it eliminates the effect of body weight and foot size variations between individuals [15]. The sensors within the mat are also larger (1.27 cm × 1.27 cm) than those of pressure plates and assess the average pressure level over each individual sensor surface. This leads to an underestimation of actual pressure values, especially in the case of pressures on small anatomical regions [71]. Coupled with tedious extraction, this factor limits the common use of such a system in the collection of plantar pressure data. However, the GAITRite walkway is extremely useful for continuous recording of a large number of steps at self-selected speeds, which could open the prospect of calculating the intra-subject variability of plantar pressures when walking under nearly ecological conditions. Similarly, given the little preparation required (no undressing and marker placement), it could be recommended in studies about plantar pressures in subjects with pathologies, or children.

Finally, the mask applied to the footprint and provided by the GAITRite system is unique. Among the multiple mask models used in other studies, some use regions of interest related to anatomical and/or functional regions with a questionable plausibility (in particular those at the forefoot [19,20,21,52] and the heel [17,20]). When using regional methods to study plantar pressure distributions, it could be suggested to identify a mask that allows an easier link between plantar pressures distribution and kinematic and kinetic foot models results. It would therefore be interesting to collectively define a mask that fits the anatomical and functional aspects of the foot by subdividing the forefoot into three segments [72].

## 5. Conclusions

Increasing gait velocity generated increased pressure peak, P*t and contact area as well as decreased peak time over the entire footprint (averaged data of fore- and rear-foot). The increase in peak pressure and P*t was not linear when pressure zones were considered except for mid-foot and medial forefoot. The results of this study suggest lower load during gait at preferred gait velocity. This was more apparent in male than female subjects. Finally, the study of the relationship between lower-limb kinematics, kinetics and plantar pressures parameters might support the notion of optimal pressure pattern at preferred velocity but it requires a reconsideration and harmonisation of the masks applied to the footprint.

## Figures and Tables

**Table 1:** Mean ± SD of subject characteristics. *p*-values are the results of a Mann—Whitney U Test by sex and effect size *r* is computed using the Rosenthal equation [28]: $r=z/\sqrt{N}$ (with a small effect when *r* ≈ 0.1, a medium effect when *r* ≈ 0.3 and a large effect when *r* ≳ 0.5).

	All Subjects *n* = 170	Women *n* = 83	Men *n* = 87	*p*-Value	*r*
Age (years)	43 ± 16	43 ± 16	42 ± 15	0.73	
Height (m)	1.72 ± 0.10	1.64 ± 0.07	1.79 ± 0.07	<0.001	−0.73
Weight (kg)	73 ± 15	66 ± 12	81 ± 13	<0.001	−0.58
BMI (kg/m^2^)	24.8 ± 4.2	24.4 ± 4.5	25.3 ± 3.7	0.04	−0.16
Leg length (cm)	91.3 ± 6.6	87.2 ± 5.5	95.3 ± 4.9	<0.001	0.65
Foot Length (cm)	25.2 ± 2.1	23.5 ± 1.1	26.7 ± 1.6	<0.001	−0.79

**Table 2:** Mean ± SD of spatiotemporal parameters for all subjects, women and men at the three different velocity conditions and their mean (appearing in a boldface type). *p*-values in the first column (S) indicate mixed-model ANOVA sex effect. In the second column (V), *p*-values indicate the result of mixed-model ANOVA velocity effects. In the third column (V × S), results of mixed-model ANOVA velocity-sex interaction are displayed (first row) and associated, when significant, with post-hoc Tukey test comparing men and women at each specific velocity (rows in line with speed data). Partial eta-squares (part. η^2^; with part. η^2^ ≈ 0.01 indicating a small effect, part. η^2^ ≈ 0.06 indicating a medium effect and, part. η^2^ ≈ 0.14 indicating a large effect) are reported as effect size (in brackets).

	All Subjects	Women	Men	*p*-Value (Part. η^2^)		
	*n* = 170	*n* = 83	*n* = 87	S	V	V × S
**Velocity (m/s)**						
MEAN	1.29 ± 0.50	1.27 ± 0.47	1.30 ± 0.52	0.59		0.002 ^a^ (0.04)
Slow	0.79 ± 0.20	0.80 ± 0.20	0.76 ± 0.20		<0.001 ^a^ (0.90)	0.93
Preferred	1.25 ± 0.19	1.24 ± 0.20	1.24 ± 0.19			>0.99
Fast	1.86 ± 0.32	1.78 ± 0.31	1.89 ± 0.31			0.02 (0.03)
**Cadence (step/s)**						
MEAN	1.87 ± 0.42	1.92 ± 0.41	1.82 ± 0.43	<0.001 (0.09)		0.40 ^a^
Slow	1.43 ± 0.21	1.48 ± 0.21	1.38 ± 0.21		<0.001 ^a^ (0.86)	
Preferred	1.86 ± 0.16	1.92 ± 0.15	1.80 ± 0.16			
Fast	2.33 ± 0.27	2.35 ± 0.25	2.28 ± 0.29			
**Step Length (m)**						
MEAN	0.67 ± 0.13	0.64 ± 0.13	0.69 ± 0.14	<0.001 (0.07)		<0.001 ^a^ (0.10)
Slow	0.54 ± 0.08	0.53 ± 0.08	0.54 ± 0.08		<0.001 ^a^ (0.89)	0.82
Preferred	0.67 ± 0.07	0.65 ± 0.08	0.68 ± 0.07			0.03 (0.05)
Fast	0.80 ± 0.09	0.76 ± 0.10	0.83 ± 0.07			<0.001 (0.15)
**Contact Time (s)**						
MEAN	0.68 ± 0.19	0.66 ± 0.18	0.70 ± 0.20	<0.001 (0.08)		0.15 ^b^
Slow	0.89 ± 0.15	0.86 ± 0.15	0.92 ± 0.15		<0.001 ^b^ (0.82)	
Preferred	0.62 ± 0.07	0.62 ± 0.06	0.67 ± 0.07			
Fast	0.50 ± 0.07	0.50 ± 0.06	0.51 ± 0.07			

^a^ Adjusted *p*-values were computed using Huynh—Feldt ε estimates. ^b^ Adjusted *p*-values were computed using Greenhouse–Geisser ε estimates.

**Table 3:** Fore- and rear-foot mean ± SD and mid-foot median [interquartile range] of plantar pressure parameters for the three different velocity conditions; computed on all subjects (*n* = 170). For the fore- and rear-foot, *p*-values reflect the effects of velocity (V, in the first column), zone (Z, in the second column) and velocity-zone interaction (V × Z, in the third column) through the results of the mixed-model ANCOVA (covariates appearing in the model are evaluated for the following values: age = 42.5 yrs). When a significant difference occurred, effect sizes are reported in brackets as partial eta-squares (part. η^2^ ≈ 0.01 indicates a small effect, part. η^2^ ≈ 0.06 indicates a medium effect and, part. η^2^ ≈ 0.14 indicates a large effect). For the mid-foot, *p*-values display the velocity effect (V, in the first column) analysed using a Friedman rank test. Effect sizes for significant effects are reported in brackets after *p*-values as average rank *r* (with r ≈ 0.1 indicating a small effect, r ≈ 0.3 a medium effect and, r ≳ 0.5 a large effect). Comparisons between medial and lateral zones can be found in the supplementary materials (Tables S2 and S3).

	Slow	Preferred	Fast	*p*-Value (Effect Size)		
				V	Z	V × Z
**Peak Pressure (%)**						
Forefoot	21.5 ± 4.4	23.6 ± 4.3	23.0 ± 3.8	<0.001 ^a^ (0.30)	<0.02 ^b^ (0.03)	<0.001 ^b^ (0.05)
Rearfoot	23.7 ± 4.3	22.4 ± 4.1	23.9 ± 3.7			
Mid-foot	3.6 [0.8–8.1]	3.3 [0.4–6.6]	2.2 [0.4–5.0]	<0.001 (0.46)		
P*t (%)						
Forefoot	47.7 ± 8.9	52.6 ±7.6	50.3 ± 5.9	<0.001 ^a^ (0.28)	<0.001 ^b^ (0.11)	<0.001 ^b^ (0.11)
Rearfoot	40.2 ± 8.5	37.9 ±7.4	42.6 ±7.2			
Mid-foot	10.9 [7.0–15.8]	8.0 [5.0–12.2]	5.9 [3.2–9.6]	<0.001 (0.58)		
**Peak Time (s)**						
Forefoot	0.71 ± 0.13	0.53 ± 0.08	0.42 ± 0.06	<0.001 ^b^ (0.30)	<0.001 ^b^ (0.86)	<0.001 ^b^ (0.14)
Rearfoot	0.34 ± 0.10	0.20 ± 0.05	0.15 ± 0.03			
Mid-foot	0.25 [0.06–0.43]	0.15 [0.03–0.28]	0.09 [0.02–0.17]	<0.001 (0.95)		
**Contact Area (cm^2^)**						
Forefoot	44.7 ± 5.7	45.9 ± 5.4	46.8 ±5.2	<0.001 ^a^ (0.26)	<0.001 ^b^ (0.18)	<0.001 ^a^ (0.03)
Rearfoot	37.6 ± 3.4	38.7 ± 3.7	39.9 ± 4.0			
Mid-foot	17.4 [12.9–22.9]	14.9 [10.1–20.4]	12.4 [8.1–18.2]	<0.001 (0.48)		

^a^ Adjusted *p*-values were computed using Huynh—Feldt ε estimates. ^b^ Adjusted *p*-values were computed using Greenhouse—Geisser ε estimates.

## Data Availability

The data presented in this study are available in Supplementary Materials S1.

## Supplementary Materials

The following supporting information can be downloaded at: https://www.mdpi.com/article/10.3390/jfmk7040106/s1, Table S1: data Leyh_VF and Tables S2–S5: Supplementary Material_Statistics and Descriptives.
