# Supplementary material for: Impact of Sex and Velocity on Plantar Pressure Distribution during Gait: A Cross-Sectional Study Using an Instrumented Pressure-Sensitive Walkway
Source: J Funct Morphol Kinesiol. 2022 Nov 28;7(4):106. doi: 10.3390/jfmk7040106 (PMC9781928; doi:10.3390/jfmk7040106)
Supplement: Supplementary file 1 [file jfmk-07-00106-s001.zip › jfmk-1988950-supplementary/Supplementary Material_Statistics and Descriptives_vf-Tables S2-S5.pdf]

Supplementary Material

Table S2. Mixed-Model ANCOVA and respective Adjusted Univariate Tests results for all **fore- and rear-foot** plantar pressure parameters.
Adjusted p-values are computed using Greenhouse-Geisser correction (GG) if ε<.75 and Huynh-Feldt correction (HF) if ε>.75. Effect sizes are assessed with partial eta-squares (part. η^2) (with part. η^2≈.01 indicating a small effect, part. η^2≈.06 a medium effect, and part. η^2≈.14 a large effect). *N*=170.

	**F-value**	***p*-value$^{Adj.}$**	**part. η^2**
**Peak Pressure (%)**			
Sex	1.45	0.23	0.01
SIDE	2.64	0.11	0.02
SIDE*Sex	0.01	0.91	0.00
VELOCITY	69.97	<0.001^{HF}	0.30
VELOCITY*Sex	0.20	0.81^{HF}	0.00
ZONE	4.63	0.02^{GG}	0.03
ZONE*Sex	0.65	0.49^{GG}	0.00
SIDE*VELOCITY	14.80	<0.001^{HF}	0.08
SIDE*VELOCITY*Sex	0.08	0.91^{HF}	0.00
VELOCITY*ZONE	8.29	<0.001^{GG}	0.05
VELOCITY*ZONE*Sex	3.13	0.04^{GG}	0.02
**P*t (%)**			
Sex	0.89	0.35	0.01
SIDE	1.42	0.24	0.01
SIDE*Sex	2.85	0.09	0.02
VELOCITY	63.95	<0.001^{HF}	0.28
VELOCITY*Sex	0.70	0.48^{HF}	0.00
ZONE	21.37	<0.001^{GG}	0.11
ZONE*Sex	2.40	0.09^{GG}	0.01
SIDE*VELOCITY	2.36	0.10^{HF}	0.01
SIDE*VELOCITY*Sex	0.44	0.63^{HF}	0.00
VELOCITY*ZONE	21.24	<0.001^{GG}	0.11
VELOCITY*ZONE*Sex	3.84	0.01^{GG}	0.02
**Peak Time (s)**			
Sex	8.60	0.004	0.05
SIDE	0.39	0.54	0.00
SIDE*Sex	3.35	0.07	0.02
VELOCITY	71.25	<0.001^{GG}	0.30
VELOCITY*Sex	3.48	0.05^{GG}	0.02
ZONE	998.24	<0.001^{GG}	0.86
ZONE*Sex	15.91	<0.001^{GG}	0.09
SIDE*VELOCITY	1.25	0.28^{GG}	0.01
SIDE*VELOCITY*Sex	6.73	0.004^{GG}	0.04
VELOCITY*ZONE	27.73	<0.001^{GG}	0.14
VELOCITY*ZONE*Sex	1.42	0.24^{GG}	0.01
**Contact Area (cm^2)**			
Sex	0.60	0.44	0.00

SIDE	2.03	0.16	0.01
SIDE*Sex	2.73	0.10	0.02
VELOCITY	57.65	<0.001^{HF}	0.26
VELOCITY*Sex	1.08	0.34^{HF}	0.01
ZONE	35.37	<0.001^{GG}	0.18
ZONE*Sex	1.48	0.23^{GG}	0.01
SIDE*VELOCITY	3.57	0.03^{HF}	0.02
SIDE*VELOCITY*Sex	0.07	0.93^{HF}	0.00
VELOCITY*ZONE	4.71	<0.001^{HF}	0.03
VELOCITY*ZONE*Sex	2.14	0.06^{HF}	0.01

Covariates appearing in the model are evaluated at the following values: Age = 42.5 yrs
^{HF} Adjusted p-values were computed using Huynh-Feldt ε estimates
^{GG} Adjusted p-values were computed using Greenhouse–Geisser ε estimates

Table S3. Friedman ANCOVA (χ^2) and Wilcoxon Matched Pairs Test (Z) results for all **midfoot** plantar pressure parameters.
Effect sizes are assessed with average rank *r* (with *r*≈.1 indicating a small effect, *r*≈.3 a medium effect and, *r*≳.5 a large effect). *N*=170.

	**χ^2-value**	**Z-value**	***p*-value**	***r***
**Peak Pressure (%)**				
SIDE		10.9	<0.001	0.84
VELOCITY	156.8		<0.001	0.46
ZONE		11.3	<0.001	0.87
SIDE*VELOCITY	446.5		<0.001	0.52
VELOCITY*ZONE	704.8		<0.001	0.83
**P*t (%)**				
SIDE		2.6	0.01	0.20
VELOCITY	197.0		<0.001	0.58
ZONE		11.3	<0.001	0.87
SIDE*VELOCITY	280.7		<0.001	0.33
VELOCITY*ZONE	724.4		<0.001	0.85
**Peak Time (s)**				
SIDE		1.8	0.07	0.14
VELOCITY	322.2		<0.001	0.95
ZONE		11.3	<0.001	0.87
SIDE*VELOCITY	653.0		<0.001	0.77
VELOCITY*ZONE	758.2		<0.001	0.89
**Contact Area (cm^2)**				
SIDE		2.2	0.03	0.17
VELOCITY	163.8		<0.001	0.49
ZONE		11.3	<0.001	0.87
SIDE*VELOCITY	245.0		<0.001	0.28
VELOCITY*ZONE	716.3		<0.001	0.84

Table S4. Plantar pressure parameters mean ± SD for lateral and medial **forefoot and rear-foot** data at the three different velocities: slow, preferred and fast. Average peak pressure and peak time as well as summed P*t and contact area are indicated for both whole forefoot and rear-foot in the latest column (Lateral + Medial Foot Surface). *N*=170.

			Foot Surface	
		**Lateral**	**Medial**	**Lateral + Medial**
**Peak Pressure (%)**				
Slow	Forefoot	21.7 ± .4.9	21.3 ± 4.8	21.5 ± 4.4
	Rear-Foot	22.2 ± 4.5	25.1 ± 4.8	23.7 ± 4.3
Preferred	Forefoot	23.6 ± .4.7	23.5 ± 4.8	23.6 ± 4.3
	Rear-Foot	20.7 ± 4.0	24.1 ± 4.4	22.4 ± 4.1
Fast	Forefoot	21.4 ± 3.8	24.5 ± 4.0	23.0 ± 3.8
	Rear-Foot	22.1 ± 3.6	25.8 ± 3.9	23.9 ± 3.7
**P*t (%)**				
Slow	Forefoot	25.2 ± 5.7	22.5 ± 5.9	47.7 ± 8.9
	Rear-Foot	17.9 ± 4.2	22.4 ± 5.0	40.2 ± 8.5
Preferred	Forefoot	27.8 ± 6.2	24.7 ± 5.9	52.6 ± 7.6
	Rear-Foot	16.6 ± 3.6	21.3 ± 4.5	37.9 ± 7.4
Fast	Forefoot	23.5 ± 5.4	26.8 ± 5.5	50.3 ± 5.9
	Rear-Foot	18.7 ± 3.7	23.9 ± 4.5	42.6 ± 7.2
**Peak Time (s)**				
Slow	Forefoot	0.67 ± 0.13	0.75 ± 0.12	0.71 ± 0.13
	Rear-Foot	0.34 ± 0.10	0.35 ± 0.11	0.34 ± 0.10
Preferred	Forefoot	0.50 ± 0.07	0.56 ± 0.07	0.53 ± 0.08
	Rear-Foot	0.20 ± 0.05	0.20 ± 0.05	0.20 ± 0.05
Fast	Forefoot	0.40 ± 0.06	0.44 ± 0.06	0.42 ± 0.06
	Rear-Foot	0.15 ± 0.03	0.15 ± 0.03	0.15 ± 0.03
**Contact Area (cm^2)**				
Slow	Forefoot	21.5 ± 3.2	23.2 ± 3.1	44.7 ± 5.7
	Rear-Foot	18.3 ± 2.0	19.3 ± 2.1	37.6 ± 3.4
Preferred	Forefoot	21.6 ± 3.0	24.3 ± 3.2	45.9 ± 5.4
	Rear-Foot	18.9 ± 2.1	19.8 ± 2.4	38.7 ± 3.7
Fast	Forefoot	21.6 ± 2.8	25.2 ± 3.0	46.8 ± 5.2
	Rear-Foot	19.6 ± 2.2	20.3 ± 2.4	39.9 ± 4.0

Table S5. Lateral, medial and lateral + medial* **mid-foot** plantar pressure parameters median [interquartile range] and mean ± SD at the three different velocities: slow, preferred and fast. *N*=170.
*Average peak pressure and peak time as well as summed P*t and contact area are indicated for whole mid-foot (Lateral + Medial).

	Median [range]			Mean ± SD		
**Foot Surface**	**Lateral**	**Medial**	**Lateral + Medial**	**Lateral**	**Medial**	**Lateral + Medial**
**Peak Pressure (%)**						
Slow	7.4 [4.7-11.0]	0.8 [0.2-1.9]	8.4 [5.7-12.6]	8.3 ± 5.0	1.4 ± 1.6	9.6 ± 5.7
Preferred	6.0 [3.9-9.0]	0.4 [0.1-1.4]	7.0 [4.5-10.1]	6.9 ± 4.3	1.1 ± 1.7	8.0 ± 5.2
Fast	4.4 [2.2-7.2]	0.3 [0.0-1.3]	5.1 [2.8-8.6]	5.2 ± 3.8	1.0 ± 1.4	6.2 ± 4.6
**P*t (%)**						
Slow	7.4 [4.7-11.0]	0.8 [0.2-1.9]	8.4 [.5.7-12.6]	8.3 ± 5.0	1.4 ± 1.6	9.6 ± 5.7
Preferred	6.0 [3.9-9.0]	0.4 [0.1-1.4]	7.0 [4.5-10.1]	6.9 ± 4.3	1.1 ± 1.7	8.0 ± 5.2
Fast	4.4 [2.2-7.2]	0.3 [0.0-1.3]	5.1 [2.8-8.6]	5.2 ± 3.8	1.0 ± 1.4	6.2 ± 4.6
**Peak Time (s)**						
Slow	7.4 [4.7-11.0]	0.8 [0.2-1.9]	8.4 [.5.7-12.6]	8.3 ± 5.0	1.4 ± 1.6	9.6 ± 5.7
Preferred	6.0 [3.9-9.0]	0.4 [0.1-1.4]	7.0 [4.5-10.1]	6.9 ± 4.3	1.1 ± 1.7	8.0 ± 5.2
Fast	4.4 [2.2-7.2]	0.3 [0.0-1.3]	5.1 [2.8-8.6]	5.2 ± 3.8	1.0 ± 1.4	6.2 ± 4.6
**Contact Area (cm^2)**						
Slow	7.4 [4.7-11.0]	0.8 [0.2-1.9]	8.4 [.5.7-12.6]	8.3 ± 5.0	1.4 ± 1.6	9.6 ± 5.7
Preferred	6.0 [3.9-9.0]	0.4 [0.1-1.4]	7.0 [4.5-10.1]	6.9 ± 4.3	1.1 ± 1.7	8.0 ± 5.2
Fast	4.4 [2.2-7.2]	0.3 [0.0-1.3]	5.1 [2.8-8.6]	5.2 ± 3.8	1.0 ± 1.4	6.2 ± 4.6
